# Anti-viral activity of culinary and medicinal mushroom extracts against dengue virus serotype 2: an in-vitro study

**DOI:** 10.1186/s12906-019-2629-y

**Published:** 2019-09-18

**Authors:** Kavithambigai Ellan, Ravindran Thayan, Jegadeesh Raman, Kazuya I. P. J. Hidari, Norizah Ismail, Vikineswary Sabaratnam

**Affiliations:** 1 Virology Unit, Infectious Disease Research Centre, Institute for Medical Research, Ministry of Health, Kuala Lumpur, Malaysia; 2 Mushroom Research Centre, Institute of Biological Sciences, Faculty of Science, University of Malaya, Kuala Lumpur, Malaysia; 3 Mushroom Research Division, National Institute of Horticultural and Herbal Science, Rural Development Administration, Eumsung, Republic of Korea; 4 Department of Food and Nutrition, Junior College Division, University of Aizu, Fukushima, Japan; 5 Virology Unit, Disease Department, National Public Health Laboratory, Ministry of Health, Sungai Buloh, Selangor Malaysia

**Keywords:** Dengue virus, Mushrooms, Anti-dengue activity, Mushroom extracts, Cytotoxicity, Plaque reduction assay

## Abstract

**Background:**

Dengue is a mosquito-borne viral infection that has become a major public health concern worldwide. Presently, there is no specific vaccine or treatment available for dengue viral infection.

**Methods:**

*Lignosus rhinocerotis*, *Pleurotus giganteus*, *Hericium erinaceus, Schizophyllum commune* and *Ganoderma lucidium* were selected for evaluation of their in-vitro anti-dengue virus serotype 2 (DENV-2) activities. Hot aqueous extracts (HAEs), ethanol extracts (EEs), hexane soluble extracts (HSEs), ethyl acetate soluble extracts (ESEs) and aqueous soluble extracts (ASEs) were prepared from the selected mushrooms. The cytotoxic effects of the extracts were evaluated by the MTT assay. The anti-DENV-2 activities of the extracts were evaluated in three different assays: simultaneous, attachment and penetration assays were perfomed using plaque reduction assays and RT-qPCR assays. The effect of the addition time on viral replication was assessed by the time of addition assay, and a virucidal assay was carried out to evaluate the direct effect of each mushroom extract on DENV-2. The chemical composition of glucans, and the protein and phenolic acid contents in the extracts were estimated.

**Results:**

We found that the HAEs and ASEs of *L. rhinocerotis*, *P. giganteus*, *H. erinaceus* and *S. commune* were the least toxic to Vero cells and showed very prominent anti-DENV2 activity. The 50% inhibitory concentration (IC_50_) values of the ASEs ranged between 399.2–637.9 μg/ml, while for the HAEs the range was 312.9–680.6 μg/ml during simultaneous treatment. Significant anti-dengue activity was also detected in the penetration assay of ASEs (IC_50_: 226.3–315.4 μg/ml) and HAEs (IC_50_: 943.1–2080.2 μg/ml). Similarly, we observed a marked reduction in the expression levels of the ENV and NS5 genes in the simultaneous and penetration assays of the ASEs and HAEs. Time-of-addition experiments showed that the highest percent of anti-DENV2 activity was observed when the mushroom extracts were added immediately after virus adsorption. None of the extracts exhibited virucidal effect. Chemical composition analysis showed that the major components in the mushroom HAEs and ASEs were glucan (beta D-glucan) and proteins, however, there was no significant correlation between the anti-dengue activity and the concentration of glucans and proteins.

**Conclusion:**

These findings demonstrated the potential of mushroom extracts as anti-dengue therapeutic agents with less toxic effects.

## Background

Dengue is a critical mosquito-borne viral disease that continues to cause substantial public health burdens in Asia and the Pacific. The reported number of dengue cases has increased over the past decade. In 2017, there were 407,199 dengue cases and 874 dengue deaths reported from countries in the Western Pacific Region [1]. In Malaysia, the number of dengue cases was 82,840 and 171 deaths, the third highest among the countries in the Western Pacific region. Dengue virus (DENV) exists in nature as four antigenically distinct but closely related virus serotypes known as DENV-1, 2, 3 and 4, which can be transmitted to humans by the mosquito species *Aedes aegypti* and *Aedes albopictus*. The clinical syndrome of DENV infection had been classified into dengue with or without warning signs and severe dengue. Attempts regarding vaccine development for dengue have been a continuous challenge for many years due to the inability of the vaccines to concurrently protect against all four antigenically distinct dengue serotypes [2, 3]. Effective antiviral therapies are currently unavailable for DENV. Dengue patients are usually managed through supportive therapies until they recover without any specific treatment measures. The search for new antiviral agents is vital for the prompt treatment of dengue infection to avoid further development of severe dengue, to control the spread of the outbreak and to enhance possible vaccination programmes in the future.

Culinary and medicinal mushrooms have great prospects in the drug and nutraceutical industries. They possess a wide range of pharmacological properties, including antimicrobial, antiviral, antitumour, anti-inflammatory, immunomodulatory, hypoglycaemic and hepatoprotective properties, and thus can be considered a functional food. Mushroom antiviral activities have been reported towards human immunodeficiency virus (HIV) using lectin isolated from *Hericium erinaceus* [4] and laccase isolated from *Clitocybe maxima* [5]. An acidic protein bound polysaccharide isolated from *Ganoderma lucidium* [6] and a protein from *Grifola frondosa* [7] inhibited the replication of the herpes simplex virus (HSV). Influenza viruses have been inhibited by ganomycins A and B from *Ganoderma pfeifferi* [8] and polysaccharides isolated from *Agaricus brasiliensis* showed antiviral activity against polioviruses [9].

In this study, five culinary and medicinal mushrooms that are commercially grown in Malaysia, *Lignosus rhinocerotis (Cooke) Ryvarden*, *Pleurotus giganteus (Berk) Karunarathna & K.D.Hyde*, *Hericium erinaceus (Bull) Persoon, Schizophyllum commune* (Fr.) and *Ganoderma lucidium* (Curtis) P. Karst were selected for in-vitro screening for their anti-dengue serotype 2 (DENV-2) activity. Two types of extraction protocols were used to isolate chemical components from selected mushrooms. Initially, hot aqueous extracts and ethanol extracts were prepared from the dry powdered mushroom fruiting body. Hot aqueous extraction was employed to obtain the polar components such as polysaccharides and proteins. The non-polar components in the ethanol extracts were isolated through solvent extraction, using hexane and ethyl acetate as solvents. Before evaluating the anti-dengue effect of the extracts, the cytotoxic properties of the extracts were identified with the 3-[4,5-dimethylthiazol-2-yl]-2,5-diphenyl tetrazolium bromide (MTT) assay. The non-cytotoxic concentrations of each extract were screened for their inhibitory effects on in-vitro dengue infection. To our knowledge, this is the first report on the anti-DENV2 activities of culinary and medicinal mushrooms.

## Methods

### Culinary and medicinal mushrooms

Five mushrooms that are known for their culinary and medicinal value were selected for this study. Four of the mushrooms were authenticated by mycologists from the Mushroom Research Centre, University of Malaya and voucher specimens were deposited in the Herbarium of University of Malaya (KLU-M). The fruiting bodies of *H. erinaceus* (KLU-M 1232) were purchased from Highland Mushroom Farm (Genting Highlands, Pahang). The fruiting bodies of *P. giganteus* (KLU-M 1227) were obtained from Nas Agro Farm (Sepang, Selangor). Fruiting bodies of *S. commune* (KLU-M 1389) were purchased from Glami Lemi Biotechnology research centre (Jelebu, Negeri Sembilan). *Ganoderma lucidium* (KLU-M 1233) was purchased in dried form from Vita Agrotech Sdn. Bhd. (Tanjung Sepat, Selangor). The fruiting bodies were sliced, freeze-dried (Christ, Germany) for 1-2 days and powdered. The *L. rhinocerotis* was purchased in freeze dried form from Ligno Biotek Sdn. Bhd. (Balakong Jaya, Selangor).

### Cells and viruses

African green monkey kidney cells (Vero, ATCC® CCL-81™) and *Aedes albopictus* clone (C6/36, ATCC® CRL-1660™) were obtained from National Public Health Laboratory, Sungai Buloh, Malaysia. Vero cells were propagated in Eagle’s minimum essential medium (Sigma aldrich, St. Louis, MO) containing 2.5% sodium bicarbonate, 1% HEPES 1 M, 1% penicillin & streptomycin and 10% heat-inactivated foetal bovine serum (FBS) (Gibco, USA). The C6/36 mosquito cell line was cultured in RPMI 1640 (Gibco, USA) supplemented with 1% sodium bicarbonate and 10% FBS. The cell growth was maintained in medium containing 2% serum concentration. The DENV-2 strain New Guinea C (GeneBank Accession No. M29095) was obtained from the Institute of Medical Research, Kuala Lumpur, Malaysia. The viruses were cultured and propagated in the C6/36 cell line with RPMI 1640 medium containing 2% FBS at 30 °C. The supernatants were collected by centrifugation after two rounds of freeze-thawing and were stored at − 80 °C for further experiments.

### Preparation of crude hot aqueous extract, ethanol extract and fractions

The hot aqueous extraction method was carried out as described previously [10]. The mushroom powder was soaked overnight in distilled water (1:20 ratio, w/v). The suspension was double boiled in a water bath for 30 min and then filtered and freeze-dried to collect the hot aqueous extract (HAE). For ethanol extraction, the mushroom powder was soaked in 80% ethanol (1:10 ratio, w/v) (Merck, Germany) for two days at room temperature. The suspension was vacuum filtered. The whole procedure was repeated twice. The pooled supernatant was evaporated under reduced pressure using a rotary evaporator (Eyela N-1000, US) to collect the ethanol extract (EE). Ten grams of EE were fractionated with 100 mL of hexane (Merck, Germany) to collect hexane soluble (HSE) and hexane insoluble extracts. One hundred millilitres of ethyl acetate (Merck, Germany) and water (1:2) mixture was added to the insoluble hexane extract to collect the ethyl acetate soluble (ESE) and aqueous soluble extract (ASE). This solvent extraction was repeated three times, the extracts were pooled, and the excess solvent was evaporated under reduced pressure using a rotary evaporator. The ASE was freeze dried. All the extracts were kept at 4 °C until further use.

### Cytotoxicity assay

Preceding the screening of the mushroom extracts for their antiviral properties, a cytotoxicity assay was carried out to identify the cytotoxic effects of the extracts on Vero cells. The cytotoxicity assay was carried out using the colourimetric MTT method using a Cell Titre 96 Non-Radioactive Cell Proliferation assay kit (Promega, USA), according to the manufacturer’s instructions. The HAE and ASE stock solutions (10,000 μg/mL) were freshly prepared in a 2% maintenance medium and filter sterilized. The EE, HSE and ESE were diluted in dimethyl sulfoxide (Amresco, USA), filter sterilized and further diluted with distilled water to obtain a 5000 μg/mL stock solution. Serial dilutions of each mushroom extract were prepared in 2% maintenance medium.

Vero cells were grown in 96-well culture plates (TPP, Switzerland) at a density of 1 × 10^5^ cells/mL. After overnight incubation, the growth medium was discarded and replaced with serially diluted HAE/ASE (250–10,000 μg/mL) or EE/HSE/ESE (12.5–500 μg/mL) in triplicate. Blank control wells with growth medium only and cell control wells containing cells with growth medium without extracts were also included in the assay. A known antiviral drug, ribavirin (Sigma Aldrich, St. Louis, MO) at concentrations ranging from 25 to 1000 μg/mL was included in the test. Microplates were incubated for 48 h at 37 °C with 5% CO_2_. The absorbance was measured at 570 nm using a 96-well plate reader (TECAN, Switzerland) which was directly proportional to the number of living cells in the wells. The 50% cytotoxic concentration (CC_50_) was estimated as the concentration of the extracts capable of decreasing the absorbance by 50% in comparison to the negative controls by probit analysis of the dose-response curve generated from the data. The maximum non-cytotoxic concentration (MNCC) was established from the concentration that showed the least cytotoxic effect towards Vero cells compared with the negative control. The concentrations below the MNCC were selected to evaluate the anti-dengue activity of the mushroom extract using a plaque reduction assay.

### Evaluation of anti-dengue activity of mushroom extracts using a plaque reduction assay

Anti-dengue assays were evaluated in three different assays, simultaneous, attachment and penetration, using plaque reduction assay [11]. During the simultaneous assay, the Vero cell monolayer was infected with 80–100 plaque forming units (PFU) of DENV-2. The plates were incubated in 5% CO_2_ at 37 °C for l h and then overlaid with 1% carboxymethylcellulose overlay medium containing various concentrations of HAE, EE, ESE or ASE below the MNCC. For the attachment assay, Vero cells were infected with 500 PFU of DENV-2 in the absence or presence of mushroom extracts. After viral adsorption for 60 min at 4 °C, both the extract and unabsorbed viruses were removed and overlaid with carboxymethylcellulose [12]. In the penetration assay, Vero cells were adsorbed with 500 PFU of DENV-2 for 1 h at 4 °C. Unbound viruses were removed, and the plates were shifted to 37 °C to allow for penetration. Cells were treated with mushroom extracts and incubated for 1 h at 37 °C. The extracts were discarded, and cells were treated with 0.1 ml of citrate-buffer (40 mM citric acid, 10 mM potassium chloride, 135 mM sodium chloride, pH 3) for 1 min to inactivate the adsorbed but not internalized viruses. Cells were washed and overlaid with carboxymethylcellulose [12]. For all the treatments mentioned above, after the addition of carboxymethylcellulose, the plates were incubated for 7 days at 37 °C, the infected cells were stained with naphthalene blue black solution, and the number of plaques was counted. The concentration of extract required to inhibit up to 50% of viral growth (IC_50_) compared with the control group were calculated by probit analysis of the dose–response curves generated from the data. The selectivity index (SI) values of each extract were calculated as SI = CC_50_/IC_50_.

The effect of the addition time of the mushroom HAE and ASE on the replication of DENV2 was determined as described previously [9]. The overlay containing the MNCC of 2000 μg/ml was then added to the Vero cell monolayer either 2 h before viral adsorption or 1, 2, 3 or 5 h after viral adsorption (80-100PFU). Percentage of inhibitory activity of the extracts was determined.

To study the direct effect of the mushroom extract on DENV-2, assays were performed according to the previously described method [13]. Virus suspensions containing 500 PFU of the DENV-2 strain were incubated with or without mushroom extract for 2 h at 37 °C. A total of 100 μl of treated viral suspension was adsorbed onto confluent Vero cells for 1 h at 37 °C. Percentage of inhibitory activity of the mushroom extract was determined.

### Effect of mushroom extract on the expression levels of dengue envelope (ENV) and non-structural protein 5 (NS5) genes

The inhibitory activities of the mushroom extracts in the simultaneous, attachment and penetration assays were further evaluated by RT-qPCR to study the expression levels of the dengue envelope (ENV) and non-structural protein 5 (NS5) genes. Vero cells were grown to approximately 90% confluency, infected with DENV-2 and cultured in the presence of 2000 μg/ml MNCC of the mushroom extracts. After 48 h post infection, the infected cells were collected by centrifugation at 1500 rpm for 10 min. Viral ribonucleic acid (RNA) was extracted from cell pellets according to the instructions provided by the Nucleospin RNA extraction kit (MACHEREY-NAGEL, Germany). The purity and concentration of the extracted RNA were assessed using a BioSpectrophotometer (EPPENDORF, Hamburg, Germany). The absorbance ratios were between 1.8–2.0 at 260/280 nm, indicating that the RNA samples were free from protein.

The extracted viral RNA was reverse transcribed into complementary DNA (cDNA) using the iScriptTM cDNA synthesis kit (Bio-Rad Laboratories, Hercules, CA, USA). Real-time PCR was carried out on CFX96 Real-Time PCR Detection System (Bio-Rad, Hercules, CA, USA). The reaction mixture contained 10 μL of SsoAdvanced SYBR Green Supermix (Bio-Rad Laboratories, Hercules, CA, USA), 2 μL of cDNA template, 7 μL of RNase/DNase free sterile water and 0.5 μL (0.25 μM final concentration) of ENV or NS5 forward and reverse primers (Bioneer Corp., South Korea) [14, 15]. The amplification was carried out with hot start activation for 5 min at 95 °C followed by 40 cycles of 10 s denaturation at 95 °C and 30 s annealing at 55 °C. Melting curve analysis was generated to verify the specificity of the PCR product. The experiments were performed in duplicate. Gene expression analysis was performed using Bio-Rad CFX Manager Software 1.6. The results were expressed as normalized fold expression compared to the virus infected cells and normalized to the reference gene of glyceraldehyde 3-phosphate dehydrogenase (GAPDH). The primer sequences of the dengue ENV, NS5 and GAPDH genes are shown in Table 1.

**Table 1:** The primer sequences of GAPDH, NS5 and ENV

Target gene	Sequence	Tm
GAPDH-F	5′ GTG GAC CTG ACC TGC CGT CT 3′	58.4
GAPDH-R	5′ GGA GGA GTG GGT GTC GCT GT 3′	58.4
ENV-F	5′ ACA AGT CGA ACA ACC TGG TCC AT 3′	58.3
ENV-R	5′ GCC GCA CCA TTG GTC TTC TC 3′	59.0
NS5-F	5′ GGA AGG AGA AGG ACT GCA CA-3′	53.8
NS5-R	5′ ATT CTT GTG TCC CAT CCT GCT 3′	54.7

The nucleotide sequences for primers were obtained from Poh et al. [14] and Kong et al. [15]

### Estimation of the chemical composition of the mushroom extracts

The chemical compositions of the mushroom extracts that exhibited anti-dengue activity were studied. Mushroom extracts were diluted in distilled water to prepare a 10 mg/mL solution. The composition of total glucan, α-glucan, β-glucan, protein and phenolic content were estimated. The amount of total and α-glucan in the mushroom extracts were evaluated using the Mushroom and Yeast β-glucan assay procedure K-YBGL 07/11 (Megazyme, Ireland). The β-glucan content was calculated by deducting the α-glucan content from the total glucan content. The Bradford assay was used to estimate the protein content and bovine serum albumin (Sigma Aldrich, St. Louis, MO) was used as a standard [16]. Folin-Ciocalteu's reagent (Sigma Aldrich, St. Louis, MO) was utilized to determine the total phenolic content by using gallic acid (Sigma Aldrich, St. Louis, MO) as a standard [17].

### Statistical analyses

Statistical analyses were conducted using SPSS version 20. All data are expressed as the mean ± standard deviation (SD). ANOVA with post hoc comparison was used to evaluate the statistically significant differences between a treated group and untreated group. The CC_50_ and IC_50_ values were calculated by probit analysis. *P*-values of less than 0.05 (*) or 0.01 (**) were defined as statistically significant.

## Results

### The yield of the mushroom extracts

To isolate as many active components from mushrooms as possible, a very extensive extraction procedure was carried out on mushroom powder. Initially, the HAE and EE were prepared from mushroom freeze-dried powder. The EE was further refined using ethyl acetate and hexane to separate the ESE and HSE, respectively. Finally, the insoluble substances were collected as the ASE. The extraction yields of the mushroom extracts are shown in Table 2. The extraction yield of the HAE was higher than that of the EE. Separation of EE produced a higher yield of ASE, followed by HSE and ESE.

**Table 2:** The percentage of extraction yield of the mushroom extracts

	HAE	EE	Separation of EE		
			ASE	ESE	HSE
*P. giganteus*	34.4	23.4	15.0	0.7	0.2
*L.rhinocerotis*	23.1	4.7	2.6	0.4	0.7
*H. erinaceus*	34.8	11.7	8.3	0.5	1.8
*S. commune*	24	13.8	8.8	0.2	3.5
*G. lucidium*	4.5	3.3	0.9	0.4	0.7

The percentage of extraction yield was calculated using the following formula: Weight of extract/Weight of mushroom powder × 100

### Cytotoxic effect and anti-dengue activity of the mushroom extracts in Vero cells

The mushroom ASEs and HAEs were less cytotoxic compared to the EEs, HSEs and ESEs (Table 3). The ASE of *P. giganteus*, *H. erinaceus* and *S. commune* showed no alteration in cell viability at concentrations up to 10,000 μg/mL. Their CC_50_ values were considered greater than 10,000 μg/mL. The HAEs of *P. giganteus*, *L. rhinocerotis*, *H. erinaceus* and *S. commune* were moderately cytotoxic; their CC_50_ values were between 3599.2 and 5171.4 μg/mL. The greatest cytotoxic effects were noted in the EEs, ESEs and HSEs; the CC_50_ values were 175.3–1967.2 μg/mL, 150.7–500 μg/mL and 60.6–500 μg/mL, respectively.

**Table 3:** Cytotoxic effects of the mushroom extract in Vero cells

	HAE	EE	ASE	ESE	HSE
*P. giganteus*	4211.8 ± 185.6**	319.5 ± 38.8**	> 10,000	171.8 ± 16.2**	163.8 ± 15**
*L. rhinocerotis*	3599.2 ± 417.8**	1967.2 ± 580.7*	3051 ± 244.6**	343.6 ± 5.3**	184.5 ± 19.2**
*S. commune*	4262.8 ± 1345.7*	> 500	> 10,000	>500	>500
*H. erinaceus*	5171.4 ± 1309.7*	817 ± 229.7*	> 10,000	150.7 ± 25.7**	60.6 ± 14.5*
*G. lucidium*	1748.1 ± 189.8**	175.3 ± 1.7**	779.1 ± 125**	>500	354.2 ± 3.6**

The cytotoxicities of the mushroom extracts were measured at the end-point (48 h) by MTT assay. The CC_50_, 50% cytotoxic concentrations capable of reducing the absorbance by 50% in comparison to the negative control cells without extract, were estimated from dose response curves of three independent experiment using probit analysis (*n* = 3). Statistical differences compared to untreated cell control group are noted with asterisk (*P < 0.05) or (**p < 0.01)

**Fig. 1:**
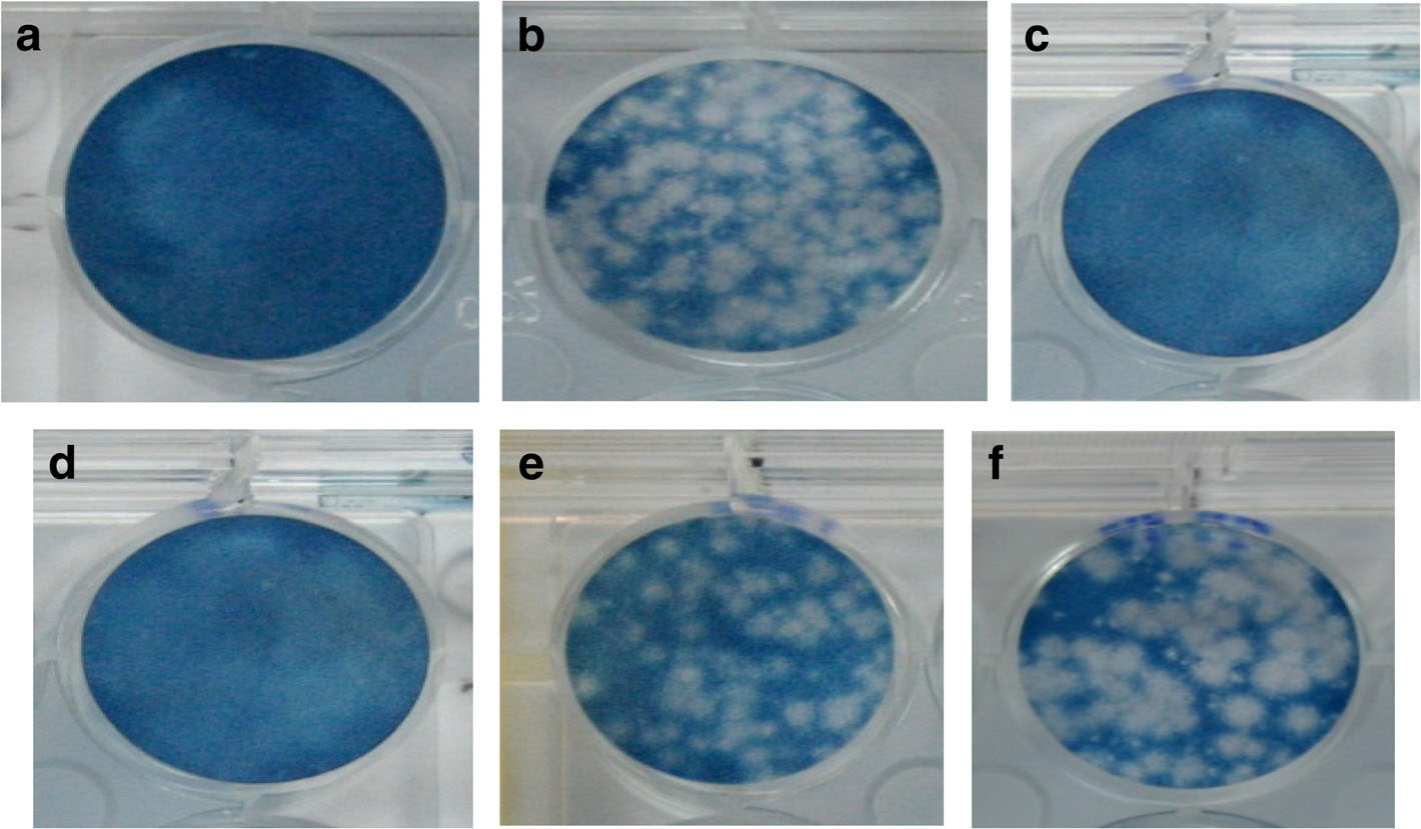
Dose dependent inhibition of *S. commune* HAE by plaque reduction assay: **a** Uninfected Vero cells, **b** Vero cells infected with DENV2 (NGC strain) (80–100 PFU), **c** Infected cell after treated with Ribavirin (250 μg/ml), **d**, **e** and **f** Infected cell after treated with *S. commune* HAE (2500 μg/ml, 1500 μg/ml and 500 μg/ml)

The anti-dengue activity was prominent in the HAEs and ASEs during the simultaneous (s) and penetration (p) assays (Table 4). The EEs, HSEs and ESEs did not show anti-dengue activity (data not shown). The most prominent anti-dengue activities were detected in the ASE of *L. rhinocerotis* (IC_50 S:_ 399.2 μg/ml; IC_50 P:_ 226.3 μg/ml), followed by *S. commune* (IC_50 S:_ 424.9 μg/ml; IC_50 P:_ 279.3 μg/ml), *H. erinaceus* (IC_50 S:_ 574.4 μg/ml; IC_50 P:_ 278.7 μg/ml) and *P. giganteus* (IC_50 S:_ 637.9 μg/ml; IC_50 P:_ 315.4 μg/ml). Higher SI values were noted in the ASEs of *S. commune* (SIs: 24.1; SI_P:_ 36), *H erinaceus* (SIs: 17.7; SI_P:_ 38.2) and *P. giganteus* (SIs: 15.7; SI_P:_ 32.3). Although *L. rhinocerotis* gave the highest IC_50_ value, due to its cytotoxic effects, *L. rhinocerotis* produced the lowest SI values during simultaneous (SIs:7.6) and penetration assays (SIp: 17.5). Weak inhibitory activity was observed from the *G. lucidium* HAE and activity was not detected in its ASE (data not shown). Figure 1 shows the dose dependent activity of *S. commune* HAE from the plaque reduction assay. We found that the SI values of the ASEs were greater than those of ribavirin during the simultaneous and penetration assays.

**Table 4:** Inhibitory activities of the mushroom HAEs and ASEs during screening, attachment and penetration assays

	Screening		Attachment		Penetration	
	IC_50_ (μg/ml)	SI	IC_50_ (μg/ml)	SI	IC_50_ (μg/ml)	SI
*P. giganteus* HAE	344.8 ± 35.4**	12.4 ± 2.2**	–	–	1731 ± 160**	2.5 ± 0.3**
*L. rhinocerotis* HAE	485.9 ± 69.5**	7.4 ± 0.4**	–	–	–	–
*S. commune* HAE	312.9 ± 14.1**	13.7 ± 4.8*	–	–	943.1 ± 70.4**	4.5 ± 0.4**
*H. erinaceus* HAE	680.6 ± 79.3**	7.8 ± 2.6*	–	–	2080.2 ± 252.7**	2.5 ± 0.3**
*P. giganteus* ASE	637.9 ± 40.3**	15.7 ± 1**	872 ± 63.2**	11.5 ± 0.9**	315.4 ± 52.4**	32.3 ± 5.7**
*L. rhinocerotis* ASE	399.2 ± 18.9**	7.6 ± 0.6**	261.2 ± 38.3**	11.9 ± 1.7**	226.3 ± 157.1	17.5 ± 8.7
*S. commune* ASE	424.9 ± 76.6**	24.1 ± 4.4**	1245.8 ± 73.7**	8.1 ± 0.5**	279.3 ± 27.3**	36 ± 3.7**
*H. erinaceus* ASE	574.4 ± 83.4**	17.7 ± 2.7**	327.6 ± 29.2**	30.7 ± 2.9**	278.7 ± 87*	38.2 ± 11.4*
Ribavirin	80.7 ± 1.8**	10.9 ± 2.8**	–	–	205 ± 15.2**	4.3 ± 0.5**

The anti-DENV2 activities of the mushroom extracts in Vero cells were evaluated in three different assays: simultaneous, attachment and penetration by the plaque reduction assay. The IC_50_ value, the concentration of extract required to inhibit 50% of virus growth compared with the virus control group, was calculated from the dose response curve of three independent experiments using probit analysis (*n* = 3). The selectivity index (SI) was calculated as the CC_50_/IC_50_. Statistical differences compared to the untreated virus control group are noted with asterisk (*P < 0.05) or (**p < 0.01)

**Fig. 2:**
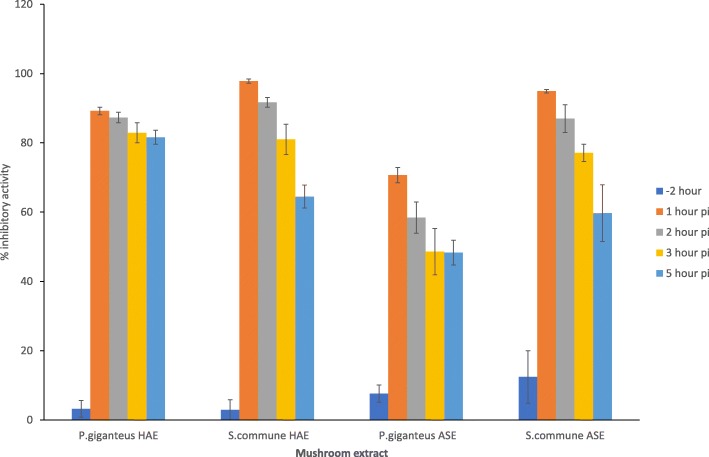
Time of addition effect of active mushroom extracts on dengue virus replication in vero cells by the plaque reduction assay. Overlay containing 2000 μg/ml of mushroom extracts were added to the Vero cell monolayer either 2 h before viral adsorption or 1, 2, 3 or 5 h after viral adsorption (80-100PFU). Percentage of inhibitory activity of extracts was determined by plaque reduction assay. Data are expressed as mean ± standard deviation of three independent experiment (*n* = 3)

The antiviral mechanisms of the mushroom extracts were further assessed by the time of addition assay. We selected *P. giganteus *and *S.commune* HAE and ASE to study this effect. We found that the extracts showed the highest percentage of inhibitory activity against DENV-2 infection when the extract was added immediately after virus adsorption (Fig. 2). The percentage of plaque inhibition decreased when the extracts were added 2 h, 3 h or 5 h after viral adsorption. Pre-treating the Vero cells with extract for 2 h prior to dengue infection did not significantly inhibit dengue plaque formation. To investigate the direct inactivating effect of the active mushroom extracts against DENV2, the virus suspension was treated for 2 h at 37 °C with the mushroom extracts. From this study, we observed that none of the tested concentrations were capable of interfering with the infectivity of DENV2, and no significant differences were detected between the dengue virus treated with extract and dengue infected control wells.

### The expression of the NS5 and ENV genes after treated with mushroom extracts

**Fig. 3:**
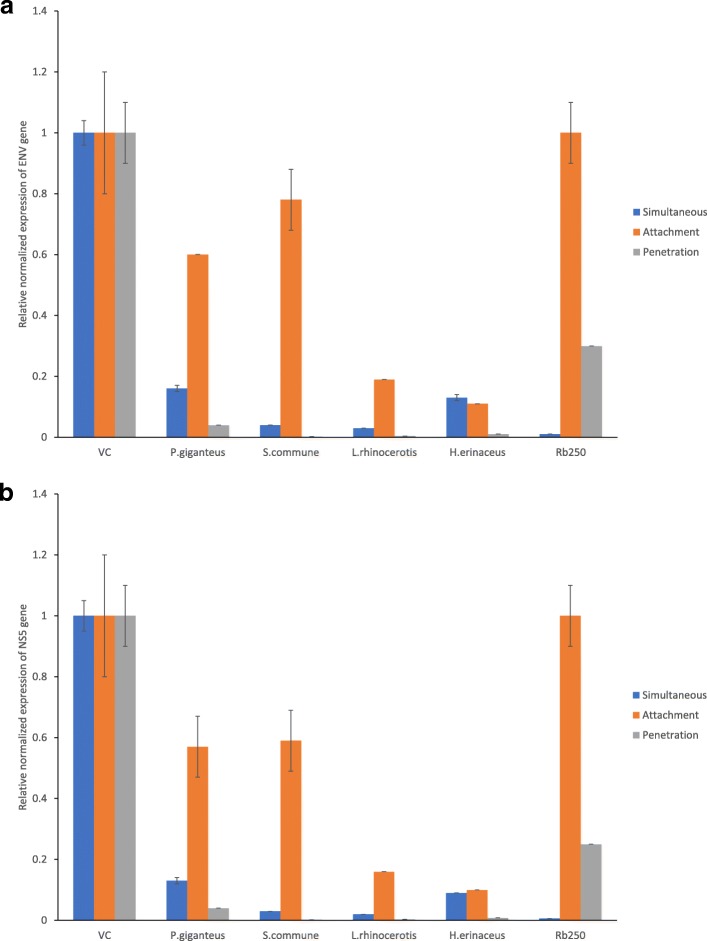
**a** Expression of ENV gene in DENV infected cell after treated with mushroom extract. **b **Expression of NS5 gene in DENV infected cell after treated with mushroom extract. DENV-2 infected Vero cells were treated with 2000 μg/ml mushroom extracts according to the protocol of simultaneous, attachment and penetration assay. After 48 h post infection, supernatants were collected to extract viral RNA of the mock infected cells, infected cells and treated infected cells. RT-qPCR was carried out to access the expression of ENV and NS5 gene. Gene expression was calculated using the algorithm provided by Bio-Rad CFX Manager Software 1.6. Results were normalized by GAPDH expression and were presented as relative normalized fold expression to virus infected cells which was defined as 1. Data are expressed as mean normalised fold expression ± SEM of two independent experiment (*n* = 2). Statistical differences compared to mock infected cells are noted with asterisk (*P < 0.05) or (**P < 0.01)

The mushroom ASEs were further selected to access their inhibitory effect on the expression of dengue ENV and NS5 genes through real-time RT-qPCR. In mock infected cells, the ENV and NS5 genes were not expressed. The upregulation of these genes was noted in virus infected cells. The results were normalized to GAPDH expression and the data are presented as the relative normalized expression to virus infected cells, which was defined as 1. All selected ASEs showed a significant reduction in the expression levels of the ENV (Fig. 3a) and NS5 genes (Fig. 3b) compared with the virus control (**P < 0.01; *P < 0.05). Similar to the plaque reduction assay, infected cells treated with ASEs showed a higher reduction in the expression of the NS5 and ENV genes in the simultaneous and penetration assays. The highest reductions were found in the ASEs of *S. commune* and *L. rhinocerotis*. In simultaneous assay, the *S. commune* ASE showed a 25-fold reduction in ENV gene expression and a 33-fold reduction in NS5 gene expression. Moreover, we observed 33 and a 50-fold reduction in ENV and NS5 gene expression in cells treated with *L. rhinocerotis* ASE, respectively. The positive control, ribavirin, gave a 100 fold reduction in ENV and a 167 fold reduction in NS5 gene expression. In the penetration assay, the *S. communae* ASE showed a 500-fold reduction in the expression of the ENV and NS5 genes. Additionally, the *L. rhinocerotis* ASE gave a 250-fold reduction in ENV gene expression and a 333 fold reduction in NS5 gene expression. For ribavirin, a significant reduction was found only in the penetration assay, and no inhibition was detected in the attachment assay. However, the level of reduction in the penetration assay was not as prominent as the mushroom ASEs, as this antiviral drug only showed a 3 to 4-fold reduction. In the attachment assay, a weak reduction was observed for the ASEs, while no inhibitory effect was detected in the HAEs. We noticed that downregulation of NS5 gene expression was more prominent than down regulation of ENV gene expression.

### Correlation between the chemical composition of the mushroom extracts and anti-dengue activity

The majority of anti-viral substances in mushroom extracts have been polysaccharides or proteins and phenolics, similar to in some natural products. We evaluated the correlation between anti-dengue activity and these components. As an anti-dengue effect was prominent in HAEs and ASEs, the chemical compositions of these extracts were studied. The main chemical composition of mushroom HAEs and ASEs was glucan and protein (Table 5). Only trace amounts of phenolic acids were detected in the HAEs and ASEs. However, the concentration and type of glucan and protein varied between the method of extraction and mushrooms specimens. Total glucan and β-glucan contents were mostly higher in the mushroom ASEs than in the HAEs, except for *L. rhinocerotis*, where the level of glucans was higher in the HAE. Moreover, the majority of the HAEs contained a higher concentration of α-glucan and protein content than the ASEs. Among all the selected mushrooms, we found that *L. rhinocerotis* contained the highest concentration of total glucan (71.8%) and β-glucan (58.4%) in its HAE, and the protein content was the highest in its ASE (27.6%). The highest concentration of α-glucan was found in the HAE of *P. giganteus* (17.6%). No significant correlation was found between the concentration of glucan or protein and anti-dengue activity, because these mushroom specimens, even those with low glucan or protein content, also exhibited prominent anti-dengue activity.

**Table 5:** The percentage of total glucan, α-glucan, β-glucan, protein and phenolic acid content in mushroom HAEs and ASEs

Extract	Total glucan^a^	α-glucan^b^	β-glucan^c^	Protein^d^	Phenolic acid^e^
*P. giganteus* HAE	27.6 ± 0.2	17.6 ± 0.1	10.1 ± 0.1	18.2 ± 2.3	0.33 ± 0.01
*P. giganteus* ASE	31.6 ± 0.4	3.5 ± 0.3	28.1 ± 0.5	12.6 ± 0.9	0.18 ± 0.01
*L. rhinocerotis* HAE	71.8 ± 4	13.5 ± 1.1	58.4 ± 1.1	16.5 ± 2.5	0.32 ± 0.06
*L. rhinocerotis* ASE	23.4 ± 0	7.8 ± 0.3	15.6 ± 0.3	27.6 ± 0.4	0.25 ± 0.03
*S. communae* HAE	4.8 ± 0.3	0.2 ± 0	4.6 ± 0.3	18 ± 1.9	0.33 ± 0.06
*S. communae* ASE	35.8 ± 0.3	1.4 ± 0.1	34.4 ± 0.4	15 ± 0.4	0.31 ± 0.05
*H. erinaceus* HAE	2.0 ± 0	0.5 ± 0.1	1.6 ± 0.1	20.6 ± 1.7	0.32 ± 0.06
*H. erinaceus* ASE	2.9 ± 1.7	0.3 ± 0.1	2.7 ± 0.1	12.7 ± 0.8	0.2 ± 0.02
*G. lucidium* HAE	9.4 ± 1.3	4.5 ± 0.4	4.9 ± 1.8	22 ± 3.7	1.28 ± 0.16
*G. lucidium* ASE	11.1 ± 0	1.3 ± 0.1	9.8 ± 0.1	19 ± 1.9	0.73 ± 0.08

Percentages of α-glucan, β-glucan, protein, phenolic content in 10 mg/ml mushroom extracts were calculated as the mean ± standard deviation of three independent experiments using the formula listed below:

^a^Total glucan: $$ \mathrm{Absorbance}\times \frac{100\ \left(\upmu \mathrm{g}\ \mathrm{of}\ \mathrm{the}\ \mathrm{D}\hbox{-} \mathrm{g}\mathrm{lucose}\ \mathrm{standard}\right)}{\mathrm{GOPOD}\ \mathrm{absorbance}\ \mathrm{for}\ 100\;\upmu \mathrm{g}\ \mathrm{of}\ \mathrm{D}\hbox{-} \mathrm{g}\mathrm{lucose}\ \mathrm{standard}}/\mathrm{weight}\ \mathrm{of}\ \mathrm{sample}\times 90 $$

^b^α-glucan: $$ \mathrm{Absorbance}\times \frac{100\ \left(\upmu \mathrm{g}\ \mathrm{of}\ \mathrm{the}\ \mathrm{D}\hbox{-} \mathrm{g}\mathrm{lucose}\ \mathrm{standard}\right)}{\mathrm{GOPOD}\ \mathrm{absorbance}\ \mathrm{for}\ 100\;\upmu \mathrm{g}\ \mathrm{of}\ \mathrm{D}\hbox{-} \mathrm{g}\mathrm{lucose}\ \mathrm{standard}}/\mathrm{weight}\ \mathrm{of}\ \mathrm{sample}\times 9.27 $$

^c^β-glucan: Total glucan – α-glucan

^d^Total protein content estimated from the bovine serum albumin standard curve (Absorbance at 595 nm = 0.001 (concentration of protein) - 0.166). % protein: concentration of protein (mg)/concentration of extract (10 mg) × 100

^e^Total phenolic content estimated from the gallic acid standard curve (Absorbance at 650 nm = 0.939 {concentration of phenolic (mM)} - 0.055). % phenolic: concentration of phenolic (mM) × 170/conc of extract (10 mg) × 100

## Discussion

Culinary and medicinal mushrooms are known as functional foods for their bioactive compounds that have various valuable impacts on human well-being. However, there are very few studies on the antiviral activities of mushroom bioactive compounds. Here, we selected a few culinary and medicinal mushrooms that are commercially available in Malaysia and evaluated their anti-viral activity against DENV. The higher yields of the HAEs and ASEs proved that there was an abundance of polar constituents, such as polysaccharides and proteins, in mushrooms. The lower yield from ethyl acetate and hexane in this study correlated with the presence of only trace amounts of fatty acids, sterols, alkaloids reported in the mushroom fruiting bodies [18–22].

The result of the cytotoxicity assay of the mushroom extracts indicated that the ASEs and HAEs were less toxic than the EEs, ESEs and HSEs. Hot water extracts prepared from mushrooms have been reported to have a less toxic effect than the ethanol extracts [18, 23]. The CC_50_ values of the ASEs and HAEs obtained from *L. rhinocerotis*, *P. giganteus, H. erinaceus* and *S. commune* were above 3000 μg/mL. The *G. lucidium* HAE and ASE showed a moderate toxic effect. The cytotoxic effect of these selected culinary and medicinal mushrooms have been studied in mouse neuroblastoma cells (N2a) and mouse embryonic fibroblast (BALB/3 T3) [23]. The CC_50_ values of the aqueous extracts of *L. rhinocerotis*, *P. giganteus* and *H. erinaceus* in N2a were 3270 μg/mL, 4070 μg/mL and 2600 μg/mL, respectively. Moreover, their toxic effects in 3 T3 cells were 5630 μg/mL for *L. rhinocerotis*, 3430 μg/mL for *H. erinaceus* and 2160 μg/mL for *P. giganteus*. A moderate toxic effect has also been reported for the *G. lucidium* aqueous extract, which was 1350 μg/mL in N2a cells and 1190 μg/mL in 3 T3 cells. These result suggest that mushroom extracts are safe for use even at high doses. Recent studies had proven that *P. giganteus* and *L. rhinocerotis* had no toxic effect on experimental rats at a daily dose of up to 5000 mg/kg and 1000 mg/kg, respectively [24, 25]. Additionally, hot aqueous extracts of *S. commune* have been safely consumed as complementary therapies for cancer patients in Japan [26]. The aqueous extract of *H. erinaceus* at a concentration of 1000 mg/kg had no toxicity in a rodent model and showed no adverse effects on the haematological and biochemical parameters [27]. Although *G. lucidium* showed a moderate toxic effect, a previous report had proven that no adverse effects were observed after oral administration of 5000 mg/kg of *Ganoderma* extract in mice [28]. The lower toxic effects of the selected mushrooms imply that there is a possibility to develop an antiviral agent for dengue virus, that is less toxic than other natural product extracts that have been tested against dengue virus, such as bioflavonoid [29], neem aqueous extract [30] and *Houttuynia cordata* aqueous extract [31].

The anti-viral activities of mushroom extracts have been extensively studied against herpes simplex virus [7, 32–34] and HIV-1 [4, 5, 35–38]. Among the selected mushrooms, *P. giganteus*, *H. erinaceus* and *S. commune* had been investigated for anti-HIV activities. Based on previous studies, the antiviral compounds of mushrooms were mainly polysaccharide [32, 35], proteoglycan [4, 36] and protein [5, 37, 38] which are water soluble.

These findings support our study, as prominent anti-dengue activities were found mostly in the HAEs and ASEs. Among the steps involved in DENV infection and replication, attachment and penetration have been considered as potential targets for the developing of antiviral drugs against the dengue virus. Here, we had proven the anti-viral potential of mushroom extracts by inhibiting of viral attachment and penetration of DENV. Extracts showed a more prominent inhibitory effect during the penetration assay tested using the plaque reduction assay and real time RT-PCR. We assume that the inhibitory activity may be initiated from attachment and gradually increases during the penetration stage. The findings presented here are in agreement with those published by other authors, who stated that the mechanism underlying the anti-viral activity of mushroom extracts may be related to the inhibition of viral adsorption and penetration [6, 9, 39]. Mushroom protein-bound glycan isolated from *Polystictus Versicolor*, PSK or Krestin, which are currently used as adjunctive therapies for various types of cancers in Japan, showed promising potential as anti-HIV agents by downregulating viral replication and promoting the upregulation of specific antiviral chemokines (RANTES, MIP-1*α*/*β*, and SDF-1*α*) known to block HIV-1 coreceptors in THP1 cells and human PBMCs [40].

In-depth study on the actual anti-dengue mechanisms of mushroom extracts is still minimal. The mechanism of the inhibition of dengue viral entry has been extensively studied on sulphated polysaccharides obtained from algae [12, 41]. Talarico and Damonte, [42] reported that the anti-DENV2 activity of carrageenan extended not only to adsorption but also to a post-adsorption event blocking the viral nucleocapsid internalization into the cytoplasm. They suggested that the fusion event leading to uncoating of the nucleocapsid and escape from the endosome was blocked, probably due to the association of carrageenan with the E virion glycoprotein. Zhang et al., [33] stated that entry inhibition occurs due to the interaction between negatively charged sulphated polysaccharides and the positively charged glycoproteins of the viral envelope, which could inhibit the adsorption of the positively charged viral particles to the negatively charged host cells. In another study, Poh et al., [14] reported that the compounds blocking the βOG (n-octyl-β-d-glucoside) pocket, which is located at a hinge between domain I and II of the envelope protein, are thought to interfere with conformational changes in the DENV envelope protein that are essential for fusion.

From these result, we found that all the selected mushroom extracts were more effective when added simultaneously with the virus, and the anti-dengue activity decreased with increasing time of infection. This shows that the inhibitory activity is dependent on the time point of exposure. These observations support our results obtained from the attachment and penetration assays, indicating that the mushroom extracts might inhibit the initial stage of DENV2 infection. Similarly, the anti-viral activity against HSV 1 and 2 using fractions obtained from β-glucan isolated from *Pleurotus tuber-regium* were more effective when added simultaneously with the virus at the time of viral infection [33]. A polysaccharide isolated from *G. lucidum* mycelia has been shown to possess better antiherpetic activity in cells with pre-treatment or treatment during the infection, instead of treatment after the infection [43]. We found that pre-treatment with mushroom extract did not protect the cells from DENV2 infection. These data suggested that there is no benefit to give the extract before infection. Mushroom extracts have no virucidal effects. This indicates that the antidengue activity was not due to an interaction with cell-free virion. Similarly, the *A. brasiliensis* aqueous extract, ethanol extract and polysaccharide also did not show virucidal activity against poliovirus [9]. Additionally, sulphated polysaccharides did not show direct inhibition of DENV activity [12].

Analysis of the chemical composition showed no significant correlation between the total glucans, protein or phenolic contents of mushroom extracts and their anti-dengue activities. The difference in anti-dengue properties between these two extracts might be due to the structural characteristics of the extracts. Ghosh et al., [44] reported that the antiherpetic properties of sulfated polysaccharides are determined by a combination of structural features such as the molecular mass, branching degree, charge density, and molecular composition of uncharged portions. Cardozo et al., [32] also recommended that a larger molecular mass and complexity of branching of sulphated polysaccharide of *Agaricus brasiliensis* are important for the inhibition of the herpes virus penetration. Based on previous findings, we could assume that the prominent anti-dengue effect of the ASE might be due to the higher molecular mass and more complex branching than that in the HAE.

## Conclusion

In conclusion, this is the first study to reveal that mushroom aqueous extracts have an in-vitro anti-dengue effect by interfering with the initial stage of DENV-2 infection, which includes the attachment and penetration of the virus through the cell membrane. Further research is needed to elucidate the structural characteristics of glucan and protein complexes that might trigger the anti-dengue effect, to prove the anti-viral effect of the mushroom on human monocytes, and suitable murine model to evaluate the oral bioavailability and metabolism of selected doses by in vivo pharmacokinetics profiling that is necessary for selecting potential anti-dengue candidate for future clinical development.

## Data Availability

The datasets generated and/or analysed during the current study are not publicly available due to being part of another study but are available from the corresponding author on reasonable request.

## Abbreviations

anti-DENV2: Anti-dengue virus serotype − 2
ASE: Aqueous soluble extract
CC_50_: 50% cytotoxic concentration
cDNA: Complementary DNA
DENV: Dengue virus
EE: Ethanol extract
ENV: Envelope gene
ESE: Ethyl acetate soluble extract
FBS: Foetal bovine serum
GAPDH: Glyceraldehyde 3-phosphate dehydrogenase
HAE: Hot aqueous extract
HIV: Human immunodeficiency virus
HSE: Hexane soluble extract
HSV: Herpes Simplex Virus
IC_50_: Concentration inhibit 50% of virus plaque
MNCC: Maximum non-cytotoxic concentration
NS5: Non-structural protein 5 gene
PFU: Plaque forming units
RNA: Ribonucleic acid
RT-qPCR: Real-time reverse transcription polymerase chain reaction
SD: Standard deviation
SI: Selectivity index
