# Correction: Anti-viral activity of culinary and medicinal mushroom extracts against dengue virus serotype 2: an in-vitro study

**DOI:** 10.1186/s12906-023-03911-2

**Published:** 2023-03-20

**Authors:** Kavithambigai Ellan, Ravindran Thayan, Jegadeesh Raman, Kazuya I. P. J. Hidari, Norizah Ismail, Vikineswary Sabaratnam

**Affiliations:** 1 Virology Unit, Infectious Disease Research Centre, Institute for Medical Research, Ministry of Health, Kuala Lumpur, Malaysia; 2 Mushroom Research Centre, Institute of Biological Sciences, Faculty of Science, University of Malaya, Kuala Lumpur, Malaysia; 3 Mushroom Research Division, National Institute of Horticultural and Herbal Science, Rural Development Administration, Eumsung, Republic of Korea; 4 Department of Food and Nutrition, Junior College Division, University of Aizu, Fukushima, Japan; 5 Virology Unit, Disease Department, National Public Health Laboratory, Ministry of Health, Sungai Buloh, Selangor Malaysia

**Correction: BMC Complement Altern Med 19, 260 (2019)**

**https://doi.org/10.1186/s12906-019-2629-y**

Following publication of the original article [1], the authors reported an error in Fig. 1. The correct figure is given below.

**Fig. 1:**
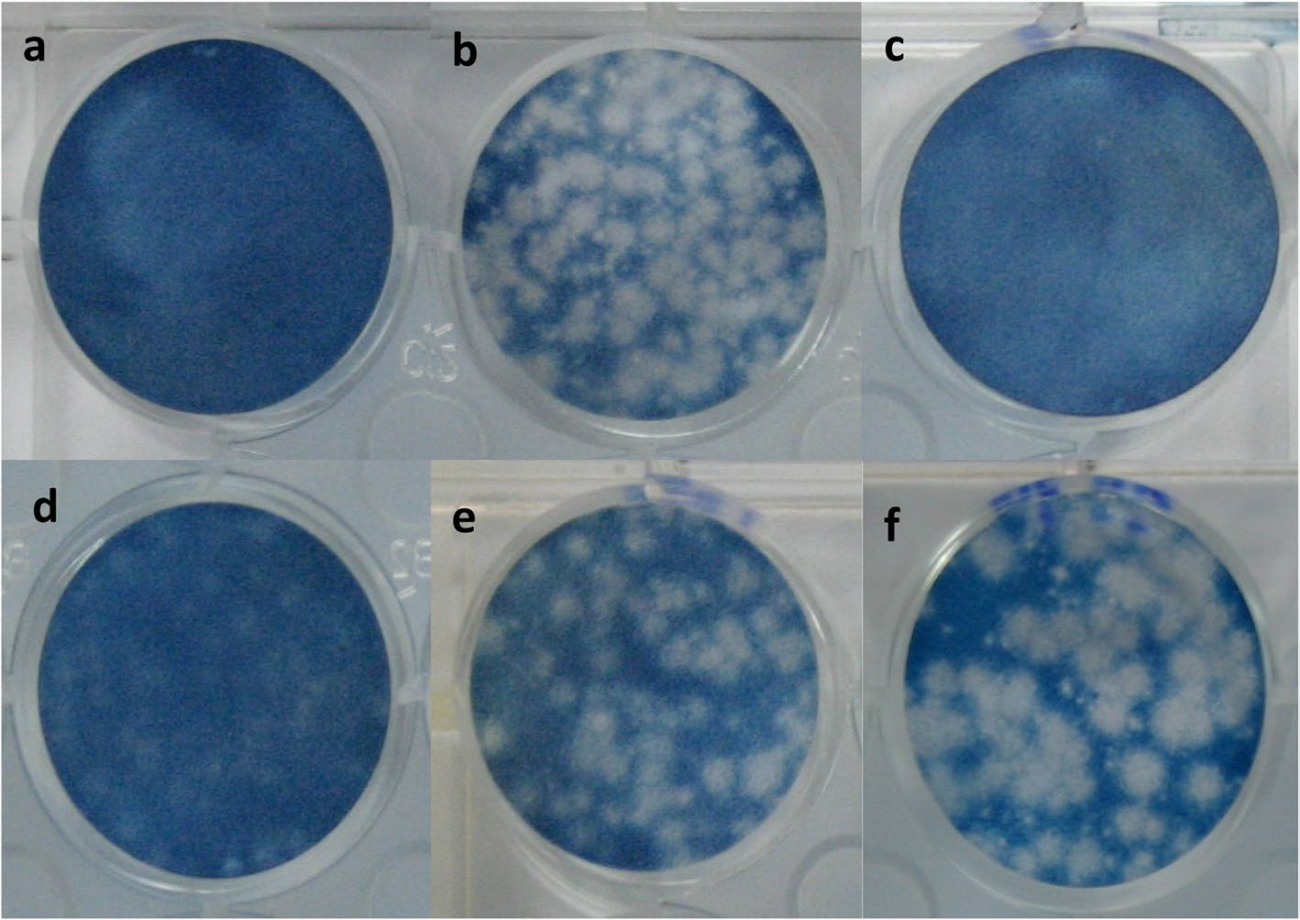
Dose dependent inhibition of *S. commune* HAE by plaque reduction assay: **a** Uninfected Vero cells, **b** Vero cells infected with DENV2 (NGC strain) (80-100 PFU), **c** Infected cell after treated with Ribavirin (250 μg/ml), **d**, **e** and **f** infected cell after treated with *S. commune* HAE (2500 μg/ml, 1500 μg/ml and 500 μg/ml)

The original article [1﻿] has been updated.
